# Effect of low-level laser therapy on quadriceps and foot muscle fatigue in children with spastic diplegia: a randomized controlled study

**DOI:** 10.1007/s10103-023-03841-y

**Published:** 2023-08-12

**Authors:** Sarah Mohamed Abdelhalim, Kamal Elsayed Shoukry, Jehan Alsharnoubi

**Affiliations:** 1 Cairo, Egypt; 2 Department of Pediatric Physical Therapy, Faculty of Physical Therapy, Cairo University, Cairo, Egypt; 3 Pediatrics Department, National Institute of Laser Enhanced Sciences, Cairo University, Cairo, Egypt

**Keywords:** Muscle fatigue, Spasticity, Lactate level, Low-level laser

## Abstract

Spastic diplegia is the most common form of cerebral palsy; children with spastic diplegia are suffering from muscle fatigue and spasticity which lead to decreasing power of muscles, impaired motor control, and many functional abilities. The effect of low-level laser (LLL) has a good result as it improves muscles pain and spasticity and in decreasing lactate levels. Forty children were selected with spastic diplegia and were divided into two groups: A and B. Group A received low-level laser treatment (LLLT) with physiotherapy treatment. Group B got physiotherapy sessions. Pain intensity was assessed by the visual analog scale (VAS) of pain which is reliable from age 5, before treatment and after 1-month follow-up. Muscle fatigue and power were assessed by maximum voluntary isometric contraction (MVIC) before treatment and after 1-month follow-up. Also, we tested blood lactate level in both groups; all evaluations were done before treatment and after 1-month follow-up. We found a significant difference between the two groups in VAS and MVIC and blood lactate level test regarding low-level therapy after 1-month follow-up. There is a good effect of low-level laser in increasing muscle power, decreasing blood lactate level, and improving pain.

## Introduction

Cerebral palsy (CP) is a clinical syndrome characterized by a persistent disorder of posture or movement due to a nonprogressive disorder of the immature brain [1]. Spastic diplegia is the most common form of cerebral palsy. It has a negative impact on blood supply, free fatty acids, the accumulation of lactic acid, and muscle glycogen depletion, which causes muscle fatigue and weakness [2]. Moreover, it results in long-term muscle tension, which limits motion and results in contractures and deformities [3]. Spasticity can cause functional problems with the activities of daily living such as eating, washing, going to the bathroom, and dressing [4].

Children with spastic diplegia typically walk slowly, and their gait patterns are characterized by excessive knee and hip flexion, implicating weakness of the ankle plantar flexors and knee and hip extensors. Due to muscle weakness and spasticity, the child needs higher force to perform the activities of daily living (ADL) and the exercises performed in a physiotherapy session. Consequently, when muscular power declines, relative strength increases, which can easily result in muscle fatigue. As a result, the child becomes exhausted from even the smallest effort and experiences pain while moving [5].

The main problem that causes muscle fatigue is that lactic acid dissociates and thus is converted into lactate which causes the accumulation of H+ ions leading to muscular acidosis; the influence of the H+ ions promotes the state of exhaustion, interrupting the breakdown of glycogen and influencing the levels of ATP which makes the child to have lower muscle power compared to typically developing child and influences on his performance of daily living activities and exercises [6].

Numerous studies have focused on ways of treatment that can be used to prevent or reduce muscle fatigue. They discovered that low-level laser is considered an important source that interacts with biological tissues, giving many physiological and therapeutical effects, including the enhancement of muscle performance [7].

Also, low-level laser was very effective in suppressing tonic muscle spasm (spasticity) in patients with cerebral palsy. It greatly improves functional training, reduces blood lactate, increases the time preceding fatigue, and increases muscular torque generation [8].

Many researches discussed the effect of low-level laser on muscle fatigue. They concluded that low-level laser therapy (LLLT) has a good effect on reducing muscle fatigue and post-exercise muscle recovery when therapy is applied before exercising [9].

The aim of our study was to examine the effect low-level laser on muscle spasm pain, muscle power, muscle fatigue, and child’s performance.

## Patient and method

### Patients

The study was performed according to the Declaration of Helsinki principles, and approval was obtained from the ethical committee of the National Institute of Laser Enhanced Sciences (NILES) review board. The study was done in the Institute Pediatric outpatient clinic in NILES and faculty of physical therapy at Cairo University, Egypt, during the period from 2021 to 2022. The current research is a randomized controlled comparative study that included 40 children of both sexes, aged 5–18 years old. All the needed information was obtained from the parents of the children.

The patients were divided randomly into two equal groups based on the treatment line. The patient selects randomly one of 40 closed envelopes, 1–20 group (A) and the 21–40 group (B).

Group A (laser group): 20 patients were subjected to LLLT with a physiotherapy treatment program daily for 5 days per week for 1 month.

Group B (control group): 20 patients were subjected to a physiotherapy treatment program daily for 5 days per week for 1 month.

### Methods

In group A, 20 patients received laser therapy over the nine points bilateraly in daily sessions for 5 days per week with physiotherapy for 1 month and were evaluated before treatment and after 1 month. In group B, patients received physiotherapy only daily for 5 days per week for 1 month and were evaluated before treatment and after 1 month.

#### The physiotherapy treatment program

First, we start with stretching exercises for all muscles of the lower limb, positioning, and mobilization for all joints of the lower limb and then Gait training between the parallel bar and using the stepper, trunk control on the ball, and balance training on the balance board.

#### Low-level laser therapy

**Fig. 1:**
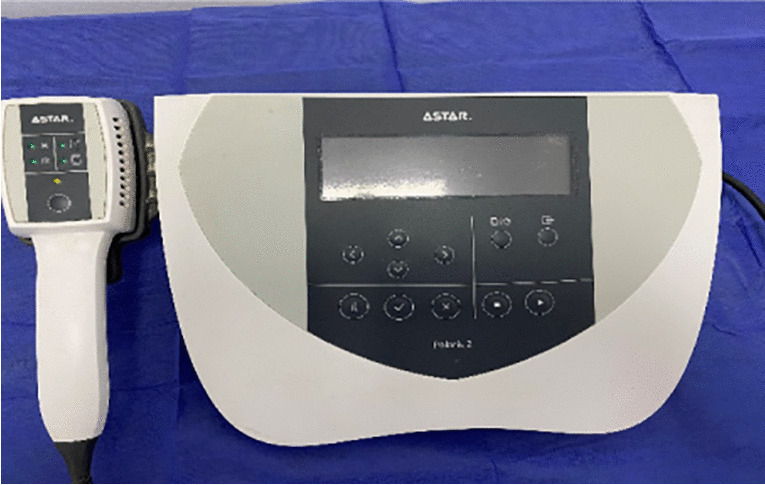
The low-level laser device

At each session, Group A received low-level laser treatment (LLLT), with infrared laser diodes, wavelength 810 nm with continuous frequency, optical output 200 mW, spot size 0.036 cm^2^, power density 5.49 W/cm^2^, and energy 30 J on each point. Energy density is 164.85 J/cm^2^. Treatment time is 1 min over each point bilateral 30 s each (Fig. 1).

**Fig. 2:**
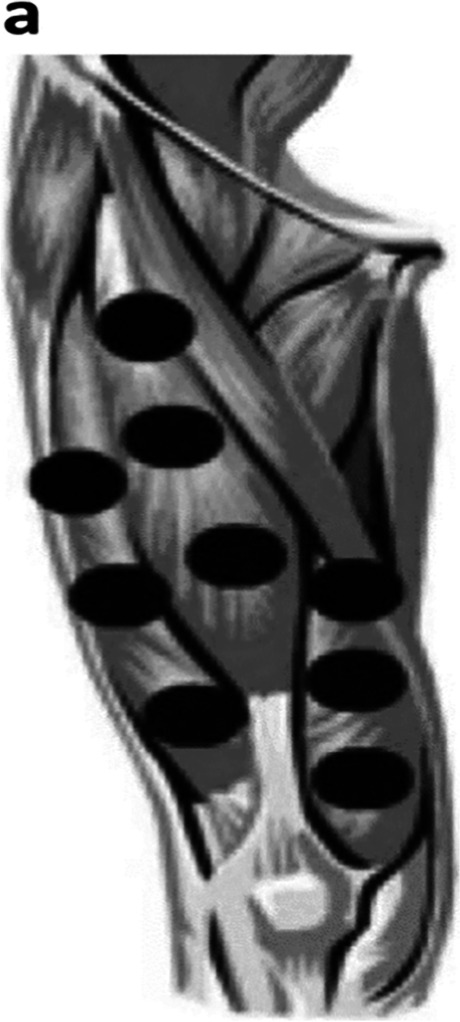
Sites of trigger points of quadriceps muscles

Nine points on quadriceps muscle between each point 1 cm (Fig. 2) and 8 points on dorsiflexors were performed daily for 5 days per week for 1 month; each session is done after stretching exercises with the traditional physiotherapy treatment program [10].

**Fig. 3:**
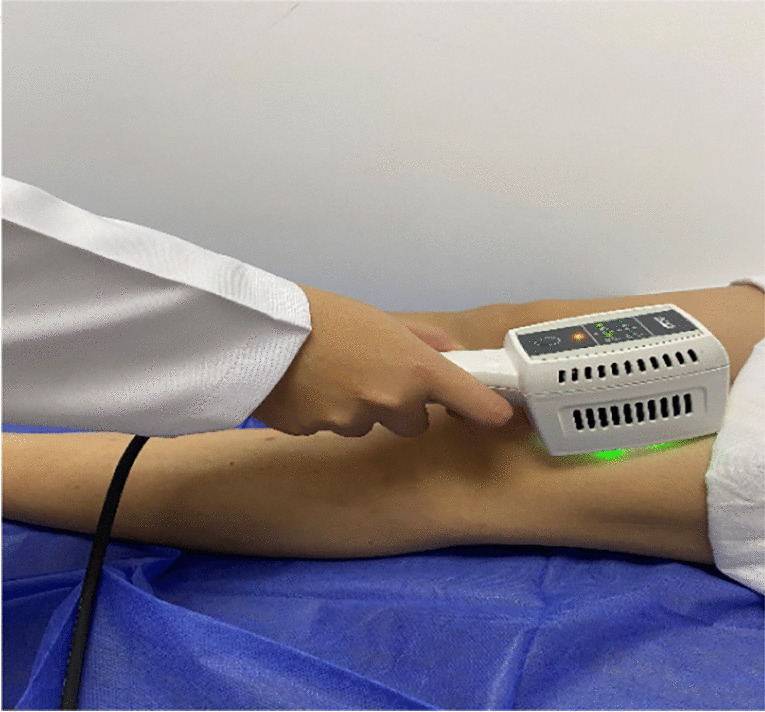
Appling laser on quadriceps muscle

LLLT was performed with the probe perpendicular over the skin (Fig. 3).

#### Inclusion criteria

Children aged from 5 to 18 years old, both sexes were chosen, all children were diagnosed with spastic diplegia grades 2 and 3 according to the Gross Motor Function Classification System (GMFCS) [11], and they were referred from pediatric neurology clinics; in addition, they have spastic quadriceps and dorsiflexors in the lower limb with any level of disability.

#### Exclusion criteria

Children who have burn or ambulated limb were excluded and any child less than 5 years, with the presence of malignant neoplastic lesion or any active infection, and children with tenotomy in the quadriceps muscle or dorsiflexors of foot.

#### Evaluation method

##### Visual analog scale (VAS)

Pain intensity assessed by the visual analog scale (VAS) of pain which consists of a horizontal line with a scale from 0 to 10, where 0 represents absence of pain and 10 represents the worst possible pain [12]. It was taken two times, at the beginning of the session after stretching exercises at the end of the session of treatment every day, 5 days per week for 1 month and after 1-month follow-up.

##### Maximum voluntary isometric contraction (MVIC) test

Maximum voluntary isometric contraction (MVIC) is a standardized method for the measurement of muscle power in patients with neuromuscular disease.

**Fig. 4:**
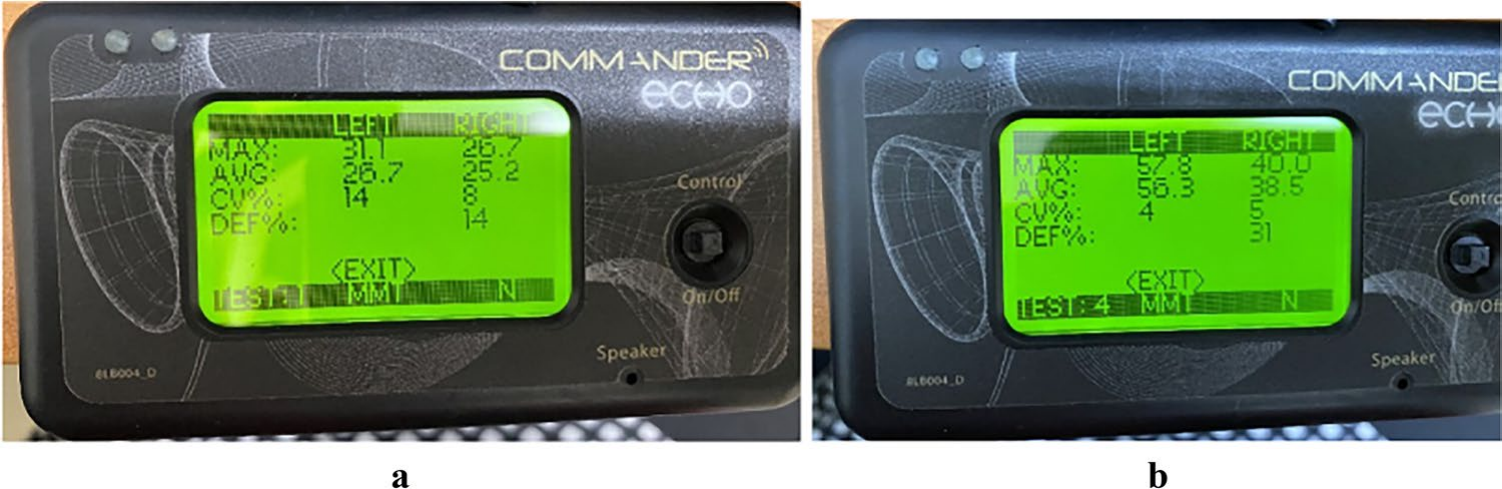
MVIC before (**a**) and after (**b**) 1-month follow-up of left and right quadriceps muscle showing improved muscular strength in group A

It was done using the Quantitative Muscle Assessment System; it is a typical technique for assessing muscle power (Fig. 4a and b) and a safe and straightforward way to evaluate muscular power as MVIC gives interval data (usually in terms of kilograms or Newton’s of force) that is more objective than manual muscle testing [13]. The reference values of both age and sex were calculated using quantile regression for each muscle group. It was taken two times, one time before treatment at the beginning of each session (after stretching exercises) and one time after treatment at the end of the session and after 1-month follow-up.

All sessions were performed at the same time of the day in the morning.

##### Lactate assessment

A blood sample was taken each session from the child two times, one time before treatment and after 1-month follow-up in both groups.

A 2 ml of venous blood was taken by a medical laboratory staff, from the nondominant arm of the patient, and a blood was collected into sterile plain tube and placed on ice packs, and samples were immediately analyzed.

#### Statistics

Statistical analysis and data were coded and entered using the Statistical Package for the Social Sciences (SPSS) version 28 (IBM Corp., Armonk, NY, USA). All the collected data were summarized using some methods of statistical analysis like mean and standard deviation. Comparisons between the groups (A and B) were made using the unpaired *t*-test. On the other hand, a before and after comparison within the same group was done using paired *t*-tests according to the following variables (visual analog scale, MVIC, and blood lactate test). All the *p*-values that are below the threshold of 0.05 were considered statistically significant [14].

## Results

The study included 40 children aged 5–18 years old from both sexes. Children were divided according to age: < 10 years and > 10 years. All children were diagnosed with spastic diplegia, and they were randomized into two groups equally based on treatment line. Data obtained from both groups pre- and post-treatment and after 1-month follow-up regarding visual analog scale, MVIC, and lactate level in blood were statistically analyzed and compared.

Regarding the age, there were no significant differences between groups (A) and (B). There was a significant difference in the VAS before and after treatment in group A *p* = 004 in children < 10 years and *p* = 0.003 > 10 years; significance were obtained in the VAS before and after treatment in group B, *p* = 0.010 in < 10 years and *p* = 0.002 > 10 years. Also, there was a significant difference between the two groups (laser and control groups) in the level of pain after 1-month treatment using VAS in the whole group, but when we divided the group according to age, there was no significance between both groups (*p* = 0.07 and 0.059 in < 10 and >10 years in groups A and B, respectively (Table 1)).

**Table 1:** Comparison between the two groups after treatment according to baseline measurements (visual analog scale, MVIC)

	Post-treatment				
	Group A		Group B		
	Mean	SD	Mean	SD	*p*-value
Vas score					
< 10	3.7	.6	4.5	.8	.074
> 10	3.8	.7	4.6	.9	.059
MVIC (doris) (RT)					
< 10	55.8	3	51.4	2.8	.006
> 10	54.4	3.4	51.6	2.6	.044
MVIC (doris) (LT)					
< 10	54.9	2.3	53.3	1.8	.236
> 10	54.3	2.6	51.7	1.9	.016
MVIC (knee ext.) (RT)					
< 10	72.8	2.4	70.7	2.1	.074
> 10	72.1	2.6	70.5	2.5	.260
MVIC (knee ext.) (LT)					
< 10	72.2	2.3	69.3	2.9	.011
> 10	71.4	2.3	69.1	1.6	.044

*p* value is significant if less than or equal to 0.05

Additionally, in the MVIC test which was applied on quadriceps of both knees, there was a significant difference between both groups after treatment with *p* = 006 and *p* = 0.044 in < 10 years and > 10 years for right dorsiflexors, respectively.

The MVIC showed no significant difference between both groups after treatment with *p* = 0.23 in < 10 years but was significant in > 10 years *p* = 0.01 for left dorsiflexors.

While MVIC was not significant in the right knee within age groups, regarding the left knee, there was a significant difference within age groups *p* = 0.01 and *p* = 0.04 in <10 years and > 10 years (Table 1 and Fig. 4a and b).

**Fig. 5:**
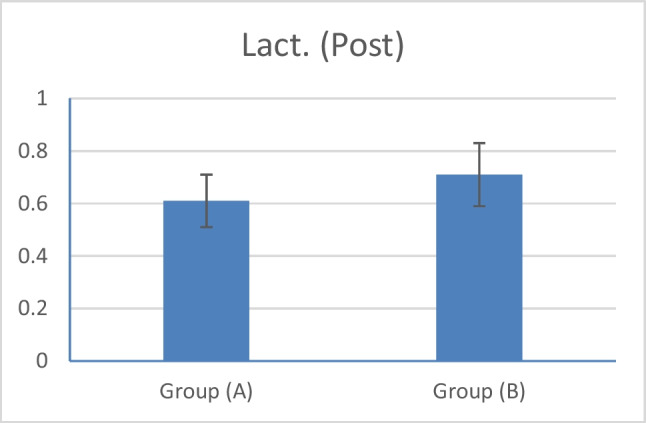
The comparison of lactate level between the two groups after treatment

Furthermore, there was a big difference in lactate levels between the two groups; in group A (laser group), there was a decrease in the level of lactate after 1-month laser therapy treatment with *p* = 0.006 (Fig. 5).

## Discussion

Muscle spasticity in children with spastic diplegia may have an impact on most of daily functions. Hence, the functional impact of reducing spasticity should be taken into account [15]. Compared to regularly growing children, they have decreased maximal muscular strength and struggle to fully activate their muscles because of recruitment issues. They should generally be thought of as being more fatigable because the majority of everyday activities are conducted at lower than maximal power [16].

Many international medical journals have several research papers talking about the interaction of low-level laser that occurs with muscular tissue. Various studies have shown that the use of low-level laser therapy (LLLT) has positive effects on delaying muscle fatigue and post-exercise muscle recovery when the therapy is applied before exercising [17]. To our knowledge, this is the first time to evaluate the effect of LLLT on the spastic and fatigued muscles (quadriceps and dorsiflexors of foot) of children with spastic diplegia.

### Sample size

Based on the previous studies by Yanying Liu and Haiping Yang (2019) indicating that the blood lactate levels pre-exercise in the placebo group were 2.26 ± 1.3 mmol/L and increased after exercise to 14.93 ± 3.0 mmol/L, while in the laser group, it was 2.37 ± 1.1 pre-exercise and increased after exercise to 12.61 ± 3.2 mmol/L to detect the difference between groups with a power of 80% and a level of significance of 5% with an effect size of 0.25, a minimum sample size of 36 participants will be needed (i.e., 18 participants in each group), and 10% will be added to compensate for possible losses; therefore, the total sample size will be 40 participants (20 participants for each group). The sample size was calculated by G*Power (version 3.1.9.2; Germany) [18].

So in our study, 40 participants (20 participants for each group) were studied. We examined the effect of low-level laser on muscle power, muscle fatigue, and child’s performance. We have used visual analog scale to test the level of pain (MVIC) and to measure the muscle power in children with neuromuscular disease and blood lactate level.

After 1-month follow-up using low-level therapy in group A, it was observed that the visual analog scale showed that there is a decrease in the level of and were evaluated before treatment and after 1 month and the child performs the exercises much easier comparing to group B. In agreement of our results, there was a study in 2019 demonstrated that low-level laser therapy when applied to the quadriceps muscle was effective in reducing knee joint pain, increasing muscle power, and consequently improving walking speed [19].

The muscle power of the quadriceps muscle and dorsiflexors of the foot for bilateral lower limbs was objectively assessed by the MVIC test. It was noted that during physical therapy sessions, children’s muscle fatigue had been delayed besides their muscle power had increased. We compared between the muscle power of the child before and after 1-month follow-up. According to the MVIC measurements, it was observed that there is an increase in muscle power and decrease in muscle fatigue of both groups more in laser group. A similar study was done in 2007 on spastic diplegia CP children; they used physiotherapy on quadriceps and triceps surae, using MVIC for evaluation, and they found that there was a greater increase in normalized force production for both quadriceps femoris and triceps surae and an increase in walking speed after training [20].

Also, our results were in agreement with a study in 2010; their findings were consistent with the observed changes in biochemical markers related to skeletal muscle recovery and the decrease in muscle fatigue [21, 22]. Another study conducted in 2016 found that the low-level laser may help in increasing the recruitment of muscle fibers, additionally increasing the onset time of the spastic muscle fatigue, and decreasing pain intensity in stroke patients with spasticity, as has been observed in healthy subjects and athletes [23]. On the contrary, another research carried out in 2020 had observed that the current photo biomodulation protocol of irradiation that was used (20 min, 808 nm, energy of 150joules) did not show any beneficial effects on quadriceps muscle performance or recovery after induction of fatigue when applied immediately after exercise [24]. This may be due to different parameters used than that of our study.

Furthermore, we found that serum lactate levels decrease in the laser group, and this was in agreement with the study in 2015, which showed a reduction in blood lactate and increased time of onset of muscle fatigue after low-level laser therapy was applied to the spastic musculature of knee extensors in individuals after a stroke [24]. In contrary to our results, a study in 2013 found that there were no differences found in the number of muscle contractions and the lactate concentration in the laser group [25].

## Conclusion

This study was the first to show the effect of LLLT on power gains on quadriceps and dorsiflexors in children with CP diplegia. LLLT is a safe, painless, and noninvasive method for children with diplegia. It effectively reduces pain and muscle fatigue, also increases muscle power, and decreases blood lactate levels during exercises.
